# Supplementary figures and images for: Age-associated decline of Coenzyme A leads to intestinal stem cells dysfunction via disturbing iron homeostasis
Source: PLoS Genet. 2025 May 30;21(6):e1011704. doi: 10.1371/journal.pgen.1011704 (PMC12151469; doi:10.1371/journal.pgen.1011704)

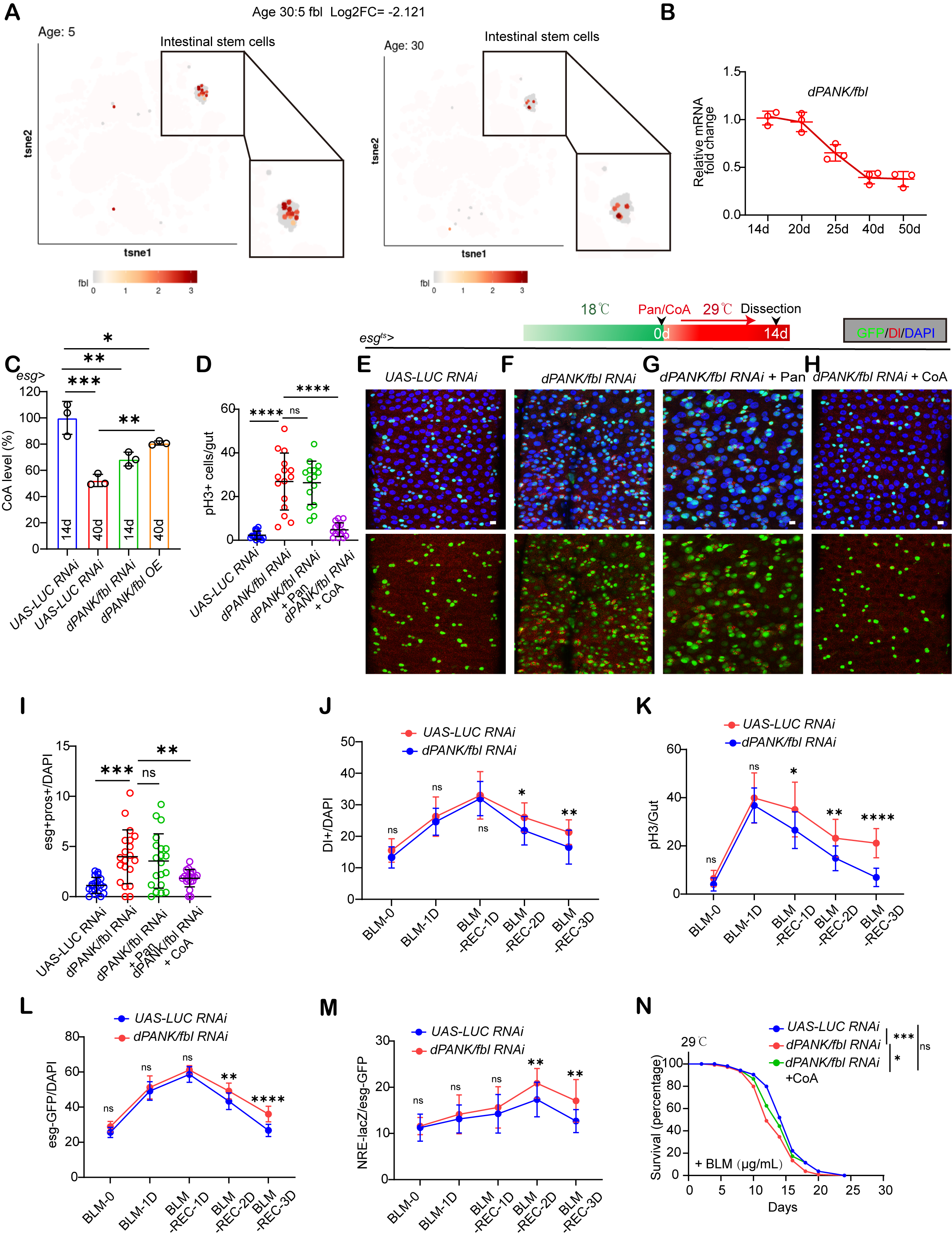

Supplement: S1 Fig — (A) Transcriptomic profiling of single-cell gene expression in dPANK/fbl between young and aged ISC populations. (B) Relative mRNA expression of dPANK/fbl from ISCs with aging. 14d is used as control and each dot represents repeating times. (C) Effects of different genotypes and age-old on CoA levels in ISCs from 14d flies with UAS-LUC RNAi (control), 40d with UAS-LUC RNAi, 14d with UAS-dPANK/fbl RNAi and 40d with UAS-dPANK/fbl driven by esg-GAL4. Each dot represents repeating times. (D) Quantification of pH3+ cells count from 14d old flies with indicated treatment. Each dot represents one midgut. (E-H) Representative photos of esg-GFP (green) and Dl+ (red) staining from flies with indicated administration from 14d flies. UAS-LUC RNAi is used as a control. (I) Quantification of the number percentage of esg+ Pros+ cells to total cells. Each dot represents one ROI from one midgut. UAS-LUC RNAi is used as a control. (J-M) Analysis of the ratio of Dl+ cells to total cells, number of ph3+ cells, ratio of esg-GFP+ cells to total cells, and ratio of NRE+ cells to esg+ cells per ROI in midguts during intestinal regeneration. UAS-LUC RNAi as a control. (N) Percentage survival rates of female flies using Canton-S as a wild-type under BLM induced chronic damage, with CoA supplementation and without it starting at 1–2-day old are presented. The numbers of quantified Drosophila: 100 for each group. Three independent experiments were conducted. The findings are based on three separate experimental trials. Each dot represents one replicate times. UAS-LUC RNAi used as a control. Scare bar: 10μm. Error bars indicate SDs. ROI size: 1.5x10^4^ μm2.Asterisks denote levels of significance: *p < 0.05, **p < 0.01, ***p < 0.001, ****p < 0.0001, and ns (not statistically significant) signifies p > 0.05. One-way ANOVA with Tukey’s multiple comparison test was used. Survival curves were analyzed using the log-rank (Mantel-Cox) test (N). (TIF) [file pgen.1011704.s002.tif]

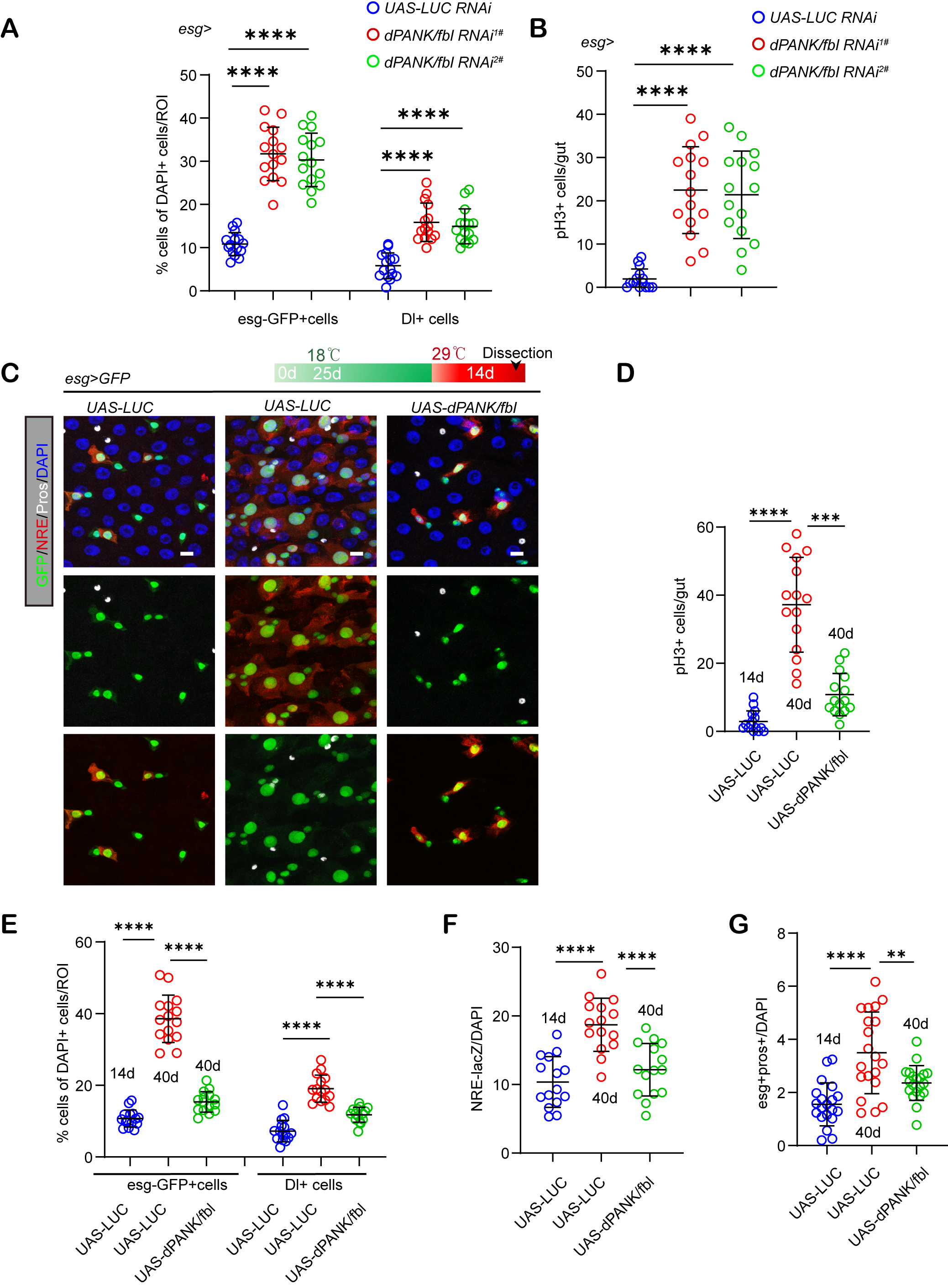

Supplement: S2 Fig — (A-B) Quantification of percentage of esg-GFP positive cells, Dl positive cells to total cells, and number of pH3 positive cells per midgut with two independent dPANK/fbl RNAi lines per ROI. Each dot represents one midgut. (C) Representative photos with esg-GFP, NRE-lacZ and Pros staining from midguts of 14d with UAS-LUC (control), 40d flies with UAS-LUC flies and 40d flies with UAS-dPANK/fbl with indicated administration. (D-G) The count of pH3 + cells percentage of esg-GFP, Dl, NRE-lacZ positive cells and esg+ pros+ cells to total cells per ROI is in S2C Fig. Each dot represents one midgut. Scare bar: 10μm. Error bars indicate SDs. ROI size: 1.5x10^4^ μm2. Asterisks denote levels of significance: *p < 0.05, **p < 0.01, ***p < 0.001, ****p < 0.0001, and ns (not statistically significant) signifies p > 0.05. One-way ANOVA with Tukey’s multiple comparison test was used. (TIF) [file pgen.1011704.s003.tif]

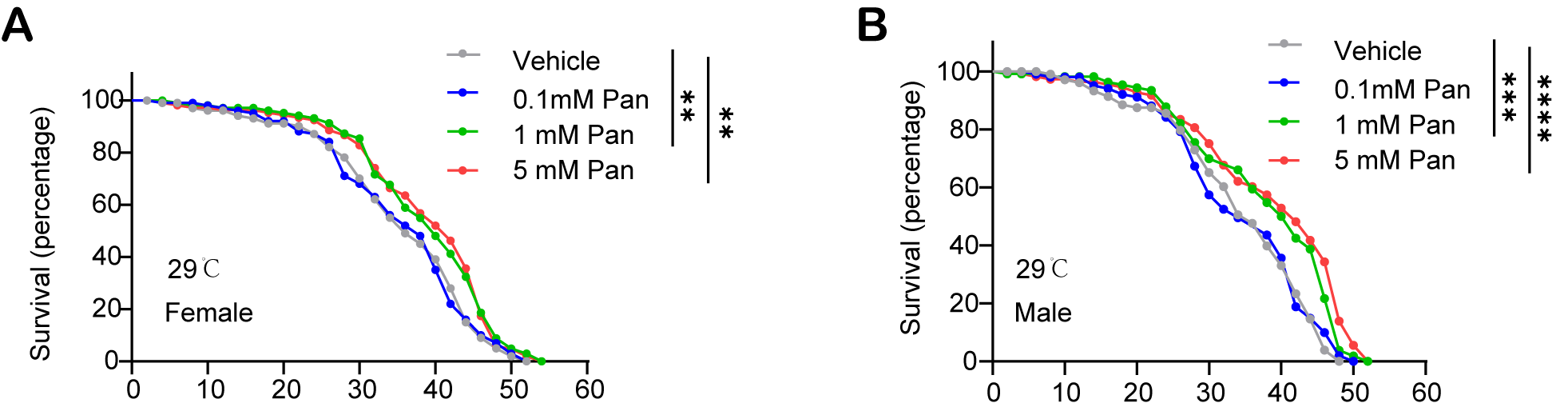

Supplement: S3 Fig — (A-B) Percentage survival rates of female flies (A) and male flies (B) and using Canton-S as a wild-type, with Pan supplementation and without it starting at 1–2-day old are presented. The numbers of quantified Drosophila: 100 for each group. Three independent experiments were conducted. The findings are based on three separate experimental trials. Each dot represents one replicate times. Error bars indicate SDs. Survival curves were analyzed using the log-rank (Mantel-Cox) test. (TIF) [file pgen.1011704.s004.tif]

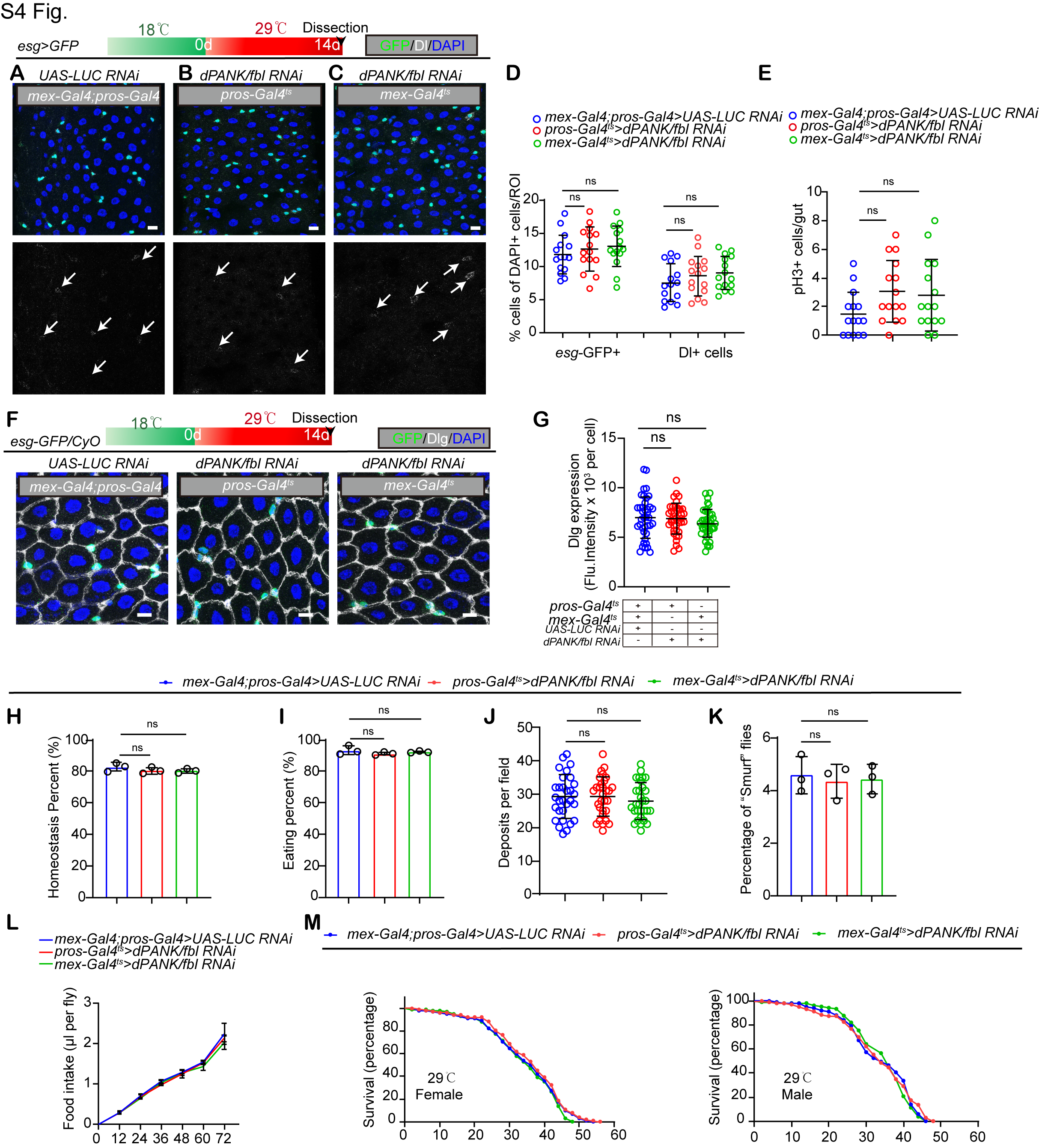

Supplement: S4 Fig — (A) Typical images from midguts stained for esg-GFP (green) and Dl (white) from 14d flies with UAS-LUC RNAi (control), pros-GAL4 driven UAS-dPANK/fbl RNAi and mex-GAL4 driven UAS-dPANK/fbl RNAi with the specified treatments indicated. (D, E) Measurement of the ratio of esg-GFP, Dl, and number of pH3 counts to total cells in the S4A-C Fig () per ROI. Each dot represents one midgut. (F, G) Representative immunofluorescence photos (F) of midguts with Dlg staining and quantification Dlg expression (G) with indicated manipulation in experiments (K) and each dot represent one ROI from R4 midguts. (H) Quantification of intestinal acid-base equilibrium in Drosophila with indicated specific GAL4. UAS-LUC RNAi line was used as a control. (I) Quantification of the eating percent of dPANK/fbl flies driven by specific GAL4. Each dot represents one replicate times. (J) Quantification of deposits number from 14d flies with UAS-LUC RNAi (control), UAS-dPANK/fbl RNAi driven with specific GAL4. Each dot represents one quantified field from 20 Drosophila. (K) Quantification of percentage of “Smurf” of indicated treatment. (L) Assessment of dietary consumption in Drosophila through the CAFE assay from 14d flies with 14d flies with UAS-LUC RNAi (control), UAS-dPANK/fbl RNAi driven with specific GAL4 of indicated treatment. Error bars show the SD of three independent experiments. Each dot represents one replicate times. (M) Percentage survival rates of female flies and male flies and using Canton-S as a wild-type, with CoA supplementation and without it starting at 1–2-day old are presented. The numbers of quantified Drosophila: 100 for each group. Three independent experiments were conducted. The findings are based on three separate experimental trials. Each dot represents one replicate times. Scare bar: 10μm. Error bars indicate SDs. ROI size: 1.5x10^4^ μm2. Asterisks denote levels of significance: *p < 0.05, **p < 0.01, ***p < 0.001, ****p < 0.0001, and ns (not statistically significant [file pgen.1011704.s005.tif]

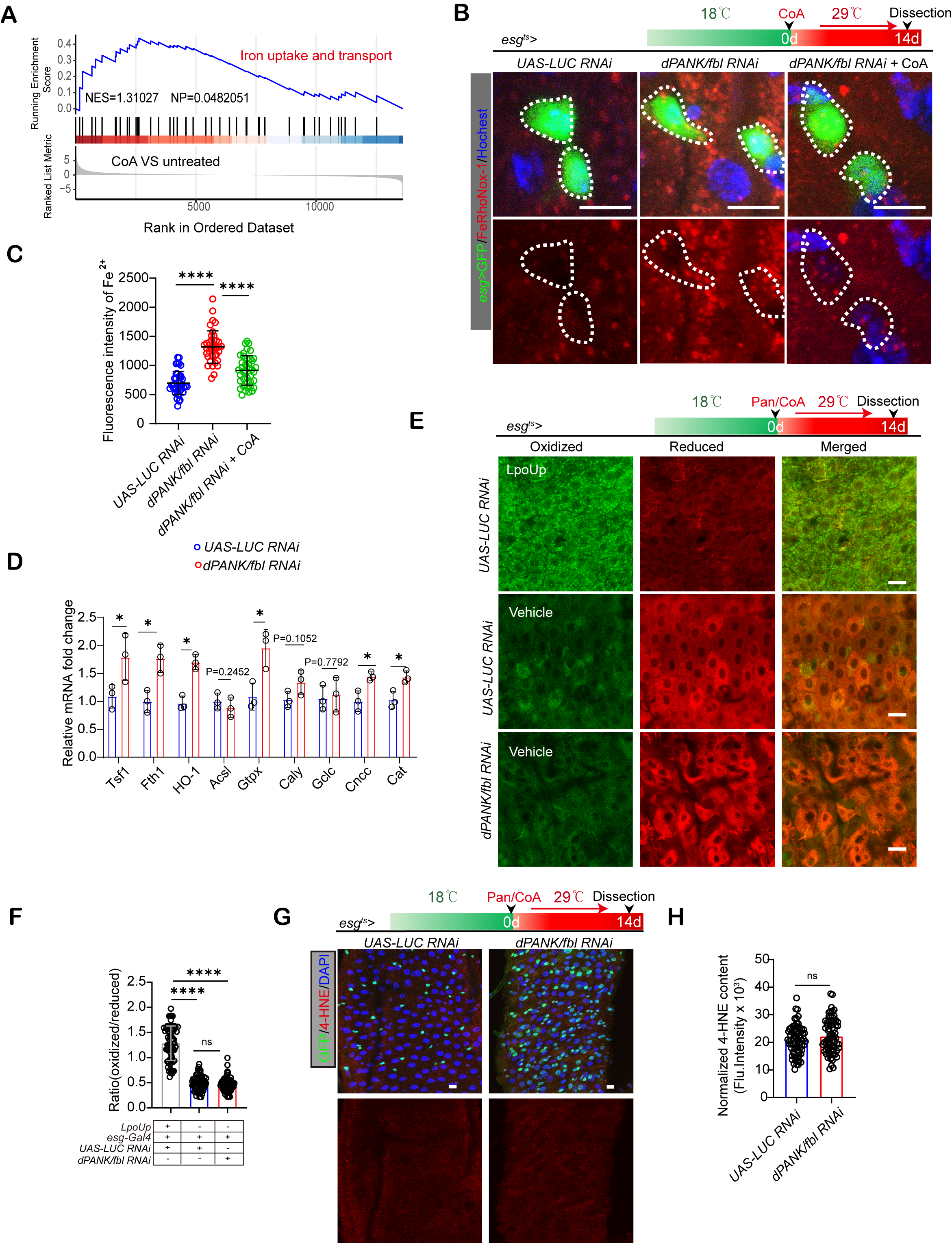

Supplement: S5 Fig — (A) Performing Gene Set Enrichment Analysis (GSEA) to compare the iron uptake and transport route between T lymphocytes subjected to CoA intervention and those that are non-treated. (B) Representative photos from midguts with FeRhoNox-1 staining (iron indicator) from 14d flies with UAS-LUC RNAi (control), and UAS-dPANK/fbl RNAi and UAS-dPANK/fbl RNAi with CoA treatment. (C) Quantification of fluorescence intensity of FeRhoNox-1 per ISCs from flies with indicated treatment. Each dot represents mean fluorescence intensity randomly chosen from 10 esg-GFP+ cells per ROI from 20 midguts. (D) Relative mRNA expression of ferroptosis associated genes in midguts of dPANK/fbl RNAi flies compared with controls (UAS-LUC RNAi) driven by esg-GAL4. Expression of control normalized to 1. n = 3 replicates. (E, F) Lipid peroxidation by BODIPY 581/591 C11 staining and quantification of C11 oxidation ratio of midguts. UAS-LUC RNAi was used as a control. (G, H) Immunostaining and quantification of 4-HNE (red) of midguts in UAS-LUC RNAi and UAS-dPANK/fbl RNAi flies Scare bar: 10μm. Error bars indicate SDs. ROI size: 1.5x10^4^ μm2. Asterisks denote levels of significance: *p < 0.05, **p < 0.01, ***p < 0.001, ****p < 0.0001, and ns (not statistically significant) signifies p > 0.05. Statistical analyses were conducted using Student’s t-tests (H), One-way ANOVA with Tukey’s multiple comparison test was used in other results. (TIF) [file pgen.1011704.s006.tif]

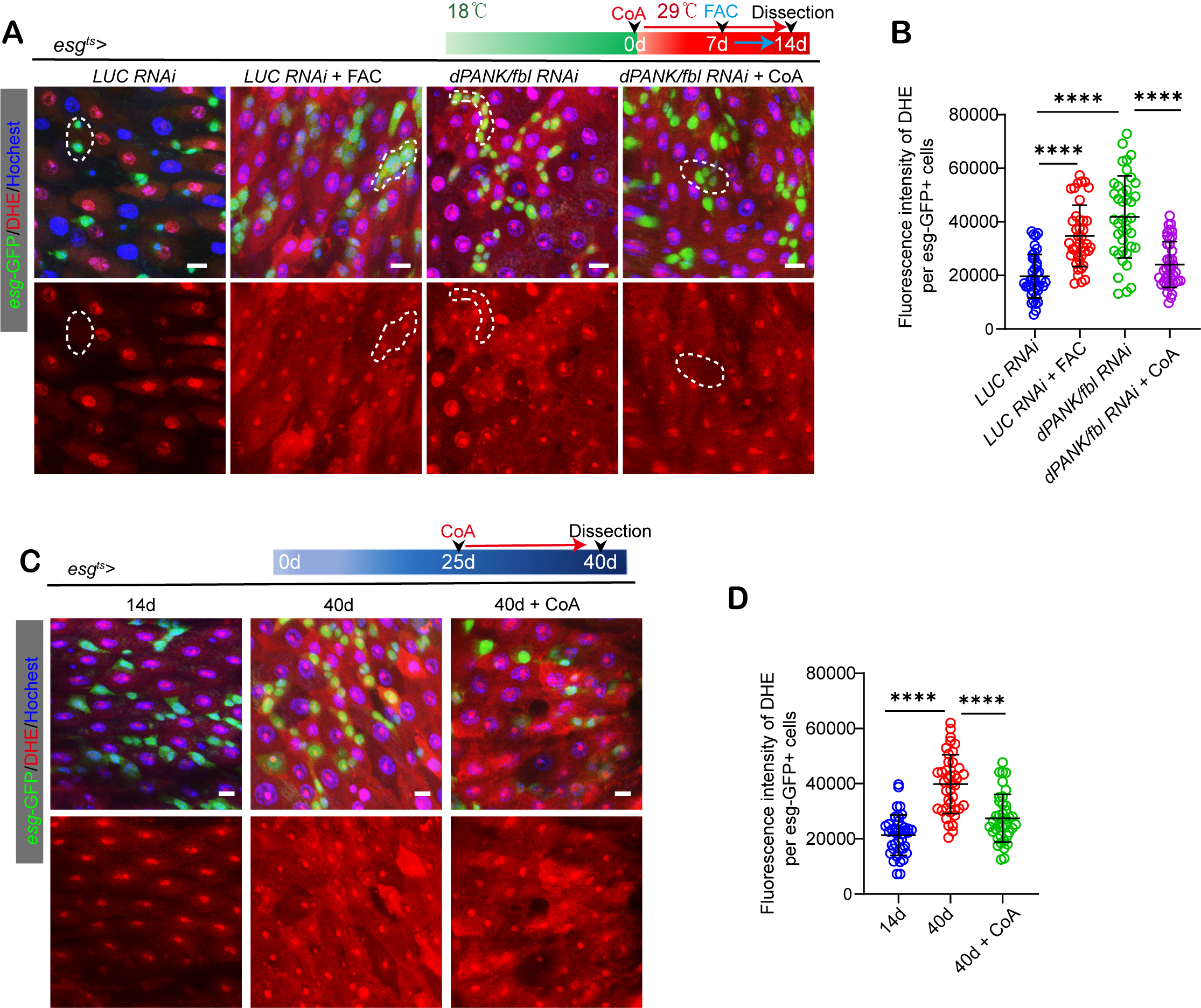

Supplement: S6 Fig — (A) Representative photos of midguts with DHE staining from 14d flies with indicated administration. UAS-LUC RNAi was used as a control. (B) Quantification of DHE fluorescence intensity per ISCs from flies with indicated administration, (C) Representative photos of midguts with DHE staining from flies with indicated administration. (D) Quantification of DHE fluorescence intensity per ISCs from flies with indicated administration, 14d flies is used as control. Each dot represents mean fluorescence intensity randomly chosen from 10 esg-GFP+ cells per ROI and total of 21 midguts. Scare bar: 10μm. Error bars indicate SDs. ROI size: 1.5x10^4^ μm2. Asterisks denote levels of significance: *p < 0.05, **p < 0.01, ***p < 0.001, ****p < 0.0001, and ns (not statistically significant) signifies p > 0.05. One-way ANOVA with Tukey’s multiple comparison test was used. (TIF) [file pgen.1011704.s007.tif]
