# Supplementary material for: Age-associated decline of Coenzyme A leads to intestinal stem cells dysfunction via disturbing iron homeostasis
Source: PLoS Genet. 2025 May 30;21(6):e1011704. doi: 10.1371/journal.pgen.1011704 (PMC12151469; doi:10.1371/journal.pgen.1011704)
Supplement: Attachment — Submitted filename: Response to Reviewers.docx [file pgen.1011704.s009.docx]

**Response to reviewers**

Dear editor and reviewers,

Many thanks for handling our manuscript. And we also extend our gratitude to three reviewers for their helpful comments in enhancing the overall quality of our article. We have addressed all the concerns reviewers highlighted and provided a comprehensive response in a point-by-point document (*italic maroon color*) that addresses each of the comments (copied in full in black). Most of these new data we have incorporated into the 6 main Figures and 6 Supplementary Figures in the revised manuscript. We have included references cited at the end of the response to support our proposed revisions. Furthermore, any changes made within the revised manuscript were highlighted in yellow.

We hope that our revision is satisfactory.

Best wishes!

Haiyang Chen

(On behalf of all authors)

**Reply to Reviewer #1：**

Reviewers' Comments:

Remarks to the Author:

Reviewer #1: In this manuscript, the authors demonstrate that reduced activity of the pantothenate/coenzyme A (CoA) pathway in aged Drosophila ISCs provides a novel and specific insight into the metabolic changes associated with aging. However, the study lacks depth in explaining how pantothenate/CoA supplementation mechanistically restructures ISC metabolism or reduces iron accumulation. Overall, this is an interesting paper and I have a few suggestions to further improve this manuscript:

*Response:*

*We sincerely appreciate your positive evaluation and the valuable comments you provided on our manuscript. Your feedback has been invaluable and has significantly enhanced the quality of our work. Our study primarily focused on the initial discovery of the crucial role of Coenzyme A in stem cell function and we have identified a downstream mechanism. Although the intermediate mechanisms are still under investigation, we will be explored in a subsequent publication and we have included a detailed discussion of the potential intermediate mechanisms in the discussion section to offer some insights. We are fully committed to addressing each of the concerns you raised and have provided a comprehensive response and solution. We believe that the revisions we have made, as detailed below, will fully address all your concerns. Thank you once again for your constructive feedback.*

1. The manuscript mentions a "screening strategy" but does not elaborate on its nature or methodology. Providing a brief description—such as whether the screening was genetic, metabolic, or otherwise—would help readers better understand the study’s approach and scope.

*Response:*

*We appreciate your valuable suggestions. In our study, we employed a targeted screening strategy to identify natural small molecule compounds that could influence ISC function. Specifically, we fed flies a diet containing these compounds and utilized an “esg-luciferase” reporter system to assess their effects on ISC activity. The luciferase activity was quantified using the Firefly Luciferase Reporter Gene Assay Kit, which allowed us to measure the impact of these compounds on ISC function. The detailed feeding technique and experimental protocol have been previously described in our laboratory's published articles [1], and we have now provided a comprehensive description of this method in both the Introduction and Methods sections of our manuscript. We believe that this additional information will help readers better understand the study’s approach and scope.*

2. The authors show that reduced CoA synthesis in young ISCs results in compromised ISC functionality. However, it remains unclear whether the regulatory effect of CoA on ISC proliferation is cell-autonomous or non-cell-autonomous. For example, would disrupting CoA synthesis in enterocytes (ECs) or enteroendocrine cells (EEs) also influence ISC proliferation?

*Response:*

*We gratefully appreciate your questions. To address whether the regulatory effect of CoA on ISC proliferation is cell-autonomous or non-cell-autonomous, we performed additional experiments using cell-type-specific RNAi lines. Specifically, we utilized pros-gal4^ts^ (EE-specific) or mex-gal4^ts^ (EC-specific) drivers to express dPANK/fbl RNAi in enterocytes (ECs) or enteroendocrine cells (EEs). The results showed that knockdown of dPANK/fbl in EEs or ECs did not significantly affect ISC proliferation, as evidenced by pH3+ cell counts and esg-GFP staining (Supplementary Fig. 4 A-E). These findings suggest that the regulatory effect of CoA on ISC proliferation is primarily cell-autonomous, indicating that CoA synthesis within ISCs themselves is crucial for their proper function.*

*Supplementary Fig. 4 A-E*

3. Similarly, it would be useful to determine if perturbing CoA synthesis in ECs or EEs affects age-related declines in intestinal functions and overall lifespan, as observed with alterations in ISCs.

*Response:*

*We appreciate your rigorous comment. In response to your suggestion, we have included statistical data on the intestinal functions and lifespan changes of flies with perturbed CoA synthesis in enterocytes (ECs) or enteroendocrine cells (EEs) in the revised manuscript (Supplementary Fig. 4 H-M). Our results showed that perturbing CoA synthesis in ECs or EEs does not significantly affect age-related declines in intestinal functions or overall lifespan. These findings further support the conclusion that the regulatory effect of CoA on ISC proliferation is ISC-autonomous.*

*Supplementary Fig. 4 H-M*

4. The data in Figure 5 show that loss of dPANK/fbl leads to a substantial increase in iron levels in both ISCs and ECs. The authors should discuss possible mechanisms driving this iron accumulation—such as altered iron import, storage, or export—and explain how these changes might impact ISC functionality and overall intestinal homeostasis.

*Response:*

*Thank you for your nice constructive remarks.* *In response to your feedback, we have added a detailed discussion of this potential mechanism in the Discussion section of our manuscript, which is highlighted in yellow.*

*While our study did not directly elucidate the mechanisms underlying iron accumulation in ISCs following the loss of dPANK/fbl, we can draw parallels with research on neurodegenerative disease models associated with PANK2 loss to investigate the link between iron dyshomeostasis and dPANK/fbl deficiency. A prominent hypothesis posits that PANK2 deficiency results in a deficiency of the active enzyme, which in turn causes an accumulation of substrates within the CoA biosynthetic pathway. Specifically, this leads to an increase in N-pantothenyl cysteine and free cysteine levels* *[2, 3]. The accumulation of cysteine can then chelate iron, potentially contributing to iron accumulation. This mechanism, if applicable in our context, could provide a plausible explanation for the observed iron accumulation in ISCs lacking dPANK/fbl.*

*Furthermore, the mitochondrial localization of PANK2 has prompted research into the role of mitochondrial dysfunction in the pathogenesis of PKAN. Notably, Paolo Santambrogio et al.**[4] have reported significant findings regarding defects in tubulin acetylation/phosphorylation within PKAN astrocytes. These defects may disrupt the regulation of vesicular dynamics, leading to improper iron delivery to the mitochondria and resulting in mislocalization of iron. The consequent cytosolic iron overload coupled with mitochondrial iron deficiency could initiate mitochondrial dysfunction. Building on these insights, we hypothesize that a similar mechanism might underlie the iron accumulation observed in ISCs in the context of dPANK/fbl deficiency. To explore this hypothesis, we plan to investigate the role of vesicular dynamics and iron trafficking in ISCs in our forthcoming studies.*

*Reducing iron levels can induce a multitude of changes, with any one of these potentially being the primary driver behind the observed phenotypic alterations. This is not unexpected, given that iron serves as a co-factor for critical components of the electron transport chain and for numerous other enzymes, including catalase and lipoxygenases, as well as for various elements within the iron regulatory system. We propose that the phenotypic outcomes in ISCs are the result of a complex interplay of multiple effects. The underlying molecular mechanisms of these effects warrant further investigation to elucidate their specific contributions to the observed phenotypes.*

5. Elevated iron levels can facilitate the Fenton reaction, thereby increasing ROS production and potentially leading to lipid peroxidation and ferroptosis. The authors should clarify whether indicators of ferroptosis—such as lipid peroxidation-induced membrane damage or changes in ferroptosis-related gene expression—are detectable in dPANK/fbl-depleted gut epithelium. Such information would provide insights into the downstream consequences of iron dysregulation in this model.

*Response:*

*We greatly appreciate your insightful suggestions. To determine whether ferroptosis contributes to the functional decline of ISCs in flies with dPANK/fbl depletion, we initially measured the expression of genes associated with the ferroptosis. As shown in Fig. Supplementary Fig. 5 D-F, the levels of genes associated with ferroptosis positive regulation that promote ferroptosis* *(Tsf1, HO-1, ferritin heavy chain 1 (FTH1) and Acsl) were significantly higher in dPANK/fbl-depleted midguts relative to WT controls, whereas the levels of the ferroptosis negative regulation signatures that suppress ferroptosis (Gtpx, Caly, Gclc, Cncc, Cat) were higher in dPANK/fbl-depleted midguts. Moreover, the level of lipid peroxidation, a biomarker indicative of ferroptosis, was comparable between dPANK/fbl-depleted midguts and WT controls. Complementary immunofluorescence quantification of 4-hydroxynonenal (4-HNE), an end-product of lipid peroxidation, further confirmed comparable oxidative lipid damage in both experimental groups. This consistent absence of ferroptotic biomarkers, despite observed differentiation defects, strongly suggests that dPANK/fbl depletion disrupts ISC-to-EC differentiation through ferroptosis-independent mechanisms. (Supplementary Fig. 5 D-F).*

*Collectively, these results suggest that the detrimental effects of dPANK/fbl deficiency on ISCs function are independent of ferroptosis.*

*Supplementary Fig. 5 D-F*

**Reply to Reviewer #2:**

Reviewers' Comments:

Remarks to the Author:

In this paper, *Liu* *et al*. identified an interesting phenotype: endogenous coenzyme A (CoA) levels in the adult fly midgut decrease with age. They proposed that this decline in CoA correlates with intestinal stem cell (ISC) hyperproliferation, a phenotype commonly observed in aging flies. Supporting this hypothesis, they demonstrated that feeding older flies with CoA could suppress ISC hyperproliferation associated with aging. Furthermore, the authors showed that knocking down dPANK/fbl, a critical gene in the CoA synthesis pathway, promotes ISC proliferation, whereas CoA supplementation counteracts this over-proliferation caused by dPANK/fbl depletion. Overall, this section of the paper is compelling, and the genetic experiments are well-designed and logically executed. Identifying Coenzyme A (CoA) as a therapeutic target to counteract ISC aging is innovative and could have broad implications for anti-aging therapies. However, the study has several significant flaws that undermine its publishability. For example, the authors concluded that CoA supplementation promotes ISC differentiation and rescues the differentiation defects caused by dPANK/fbl depletion. Unfortunately, the data presented in Figures 2 and 3K-P contradict this conclusion, as discussed in the following comments. Additionally, the conclusions drawn from Figures 5A-B, 5E-F, and 6A-B are problematic. Unfortunately, because of these shortcomings, we cannot recommend this paper for publication in PLOS Genetics in its current form. However, the topic is significant, and with substantial revisions (including new data), to address these issues the paper could be a valuable addition to the literature on stem cell functions and aging. Detailed comments are provided below.

*Response:*

*Thank you very much for your thorough review and constructive feedback on our manuscript. We appreciate your recognition of the innovative aspects of our work, particularly the identification of Coenzyme A (CoA) as a potential therapeutic target to counteract ISC aging, and the significance of our findings for anti-aging therapies.*

*We are grateful for your detailed comments and acknowledge the concerns you have raised regarding the interpretation of our data, especially in relation to Figures 2, 3K-P, 5A-B, 5E-F, and 6A-B. We understand the importance of presenting accurate and consistent results that support our conclusions, and we agree that there are areas in our manuscript that require clarification and strengthening.*

*We are committed to addressing these issues thoroughly. We revised the manuscript by re-evaluating the data, clarifying the figures, and providing a more rigorous interpretation of the results. We included additional experiments to support our conclusions and ensure that our findings are presented in a manner that is both accurate and compelling. We firmly believe that the revisions we have made, as outlined in detail below, have adequately addressed the concerns raised and further reinforced our conclusions.*

Major points:

1. As mentioned, the data shown in Figures 2 and 3K–P contradict the conclusion that CoA supplementation promotes ISC differentiation. If CoA administration indeed promotes ISC differentiation, there should be an increase in esg+mex+(Figures 2B-E), esg+pros+ (Figures 2F-I), and NRE-lacZ+ (Figures 2F-H, J) cells in the “40d+CoA” group compared to the “40d” group. Referencing typical pro-differentiation gene phenotypes (e.g., Notch overexpression) from previous publications might be helpful. However, the observed phenotype suggests the opposite, potentially indicating that CoA supplementation impairs ISC self-renewal (proliferation) and regenerative capacity. A similar misinterpretation applies to Figures 3K–P, where the data suggest that CoA blocks dPANK/fbl RNAi-induced pro-mitotic effects. Additional data are needed to rule out this alternative interpretation.

*Response:*

*Thank you for your insightful comments and for highlighting the potential discrepancies in the interpretation of our data. We understand your concern regarding the contradiction between the observed data and our initial conclusion regarding the effect of CoA supplementation on ISC differentiation.*

*Previous studies have shown aging Drosophila midgut displays a failure of ISC-to-EC differentiation and promotes ISC-to-EE differentiation compared with young flies, which is characterized by abnormal accumulation of esg-GFP, pre-EEs(pro+esg+), pre-ECs(mex+esg+) and NRE-lacZ positives cells[5, 6]. Our result showed CoA administration remarkably decreased the accumulation of esg-GFP, pre-ECs, NRE-lacZ seen in old flies. Thus, it is rational to conclude that CoA administration indeed promotes ISC-ECs differentiation from old flies. Thus, to more accurately describe these results, we have changed the term “hindered differentiation" to "hindered ISC-to-EC differentiation”.*

*As for results in Figures 3K–P from young flies, we admitted that dPANK/fbl RNAi may promote or inhibit ISC differentiation according to these current results. To rule out these two possibilities, we have now presented data in the following paragraphs:*

*Flipout line (yw; hsFLP; Act.FRT-CD2-FRT.GAL4, UAS-GFPS65T/CyO) is an excellent method to study ISC differentiation ability.* *Results of flipout showed that depletion of dPANK/fbl resulted in significantly larger clones compared to wild-type (WT) controls (Fig 3I-3K). Immunostaining revealed a marked increase in Dl-positive and pros-positive cells within dPANK/fbl RNAi clones following BLM-induced clone induction (ACI), further suggesting disrupted lineage commitment (Fig 3I, 3J, 3L and 3M).To further assess the impact of dPANK/fbl loss on ISC differentiation directionality, we quantified the munber of mature ECs (esg⁻Pros⁻ and polyploid) and EEs (esg⁻Pros⁺). dPANK/fbl-depleted midguts exhibited a reduced population of mature ECs but an elevated EE count relative to WT controls (Fig 3N, 3O). Consistent with this, RT-qPCR analysis demonstrated a significant downregulation of EC-specific enzymatic markers in dPANK/fbl-deficient flies (Fig 3P), reinforcing the impairment of terminal EC differentiation.*

*Fig. 3 I-P*

*Given the essential role of dPANK/fbl in EC production, we hypothesized that its depletion would compromise gut regenerative capacity. Post-injury analysis revealed defective midgut regeneration in dPANK/fbl RNAi flies, characterized by persistent accumulation of NRE-lacZ⁺ cells and attenuated reduction of esg-GFP⁺ ISC populations compared to pre-injury levels (S1J-S1M Fig). Furthermore, dPANK/fbl-deficient flies displayed significantly reduced survival rates under chronic BLM-induced damage, a phenotype partially rescued by CoA supplementation (S1N Fig).*

*Supplementary Fig. 1 J-N*

*Notch signaling in the stem cell daughter cell is important for EC fate but is kept off or low when EE fate specification occurs. Inhibition of Notch signalling has been shown to promote both ISCs renewal and EE differentiation. In contrast, increasing Notch activity by expression of the Notch intracellular domain (NICD) decreased the number of ISCs and pros+ cells[7]. Our results from dPANK/fbl depletion share comparable phenotypic characteristics with Notch inhibition (higher number of ISCs and pros cells). And phenotype of CoA administration closely resembles those of Notch overexpression (lower number of ISCs and pros+ cells). Thus, we thought CoA promotes ISC-to-EC differentiation.*

*(source data from Li He et al, Nature, 2018)*

*We believe that these additional data will clarify the role of CoA in ISC differentiation and address the concerns you raised. We are confident that these revisions will strengthen the manuscript and provide a more accurate interpretation of our findings.*

2. The immunofluorescence data shown in Fig. 5A-B and 5E-F, and Fig. 6A-B are sloppy, and the conclusions made on them are unreliable. How did the authors determine that the observed increase in RhoNox-1 in ISCs was not due to technical inconsistencies in staining across parallel groups? Notably, dPANK/fbl RNAi guts exhibit significantly higher overall RhoNox-1 levels. Additionally, if dPANK/fbl functions specifically in ISCs, why does dPANK/fbl knockdown increase RhoNox-1 both autonomously and non-autonomously? This requires clarification.

*Response:*

*Thank you for your careful consideration of our manuscript and for highlighting the concerns regarding the immunofluorescence data presented in Figs. 5A-B, 5E-F, and 6A-B. We sincerely appreciate your feedback and understand the importance of addressing the reliability of our conclusions.*

*Regarding the higher overall RhoNox-1 levels in dPANK/fbl RNAi guts, we believe that these EEs or ECs are descendants of stem cells with dPANK/fbl depletion. These EEs or ECs inherit iron dyshomeostasis phenotype from their mother stem cells, which could account for the elevated RhoNox-1 expression. Moreover, published study have found esg^ts^> TER94^V22^ could activate p ROS in surrounding ECs relative to control midguts, and found ISC tumor growth also activated p38 in surrounding ECs relative to control midguts bearing no tumors [8, 9]. Thus, it is possible that dPANK/fbl depletion in ISCs could increase RhoNox-1 non-autonomously.*

*(source data from* *Fuli Liu et al, J Genet Genomics, 2022)*

*(source data from* *Parthive H Patel et al, Nature Communications, 2019)*

*To further address your concerns about potential technical inconsistencies in staining, we have taken the following steps in the revised manuscript:*

*We have included additional iron indicator lines—UAS-Fer1HCH-GFP lines driven by esg-GAL4—to ensure the robustness of our staining across parallel groups (Fig. 5 A-B). These indicators are strongly upregulated in the ISCs of dPANK/fbl-RNAi flies, and their levels decrease with CoA supplementation, which aligns with our RhoNox-1 staining results.*

*Fig. 5 A-B*

*To further investigate the relationship between ROS and CoA, we have incorporated another ROS reporter line, GstD1-GFP, to measure ROS levels in ISCs of dPANK/fblRNAi flies (Fig. 6 A-B). We observed that ROS levels were significantly higher in ISCs of these groups, which is consistent with our DHE staining results.*

*Fig. 6 A-B*

*We trust that these revisions and the inclusion of additional data will provide the necessary clarification and support for our conclusions. We are grateful for your guidance.*

3. Following the above point #2, While using fluorescence intensity quantification to demonstrate significant differences among groups is a standard approach, it raises ethical concerns. Specifically, were the images selected arbitrarily? This concern is particularly relevant when phenotypes are subtle and quantification is necessary to distinguish them. The authors should provide details about the image selection process and declare how the images were chosen for quantification.

*Response:*

*Thank you for raising this important ethical concern regarding the selection of images for fluorescence intensity quantification. We understand the necessity of transparency in the image selection process, especially when phenotypes are subtle.*

*In response to your query, all immunofluorescence images were acquired using a Leica TCS-SP8 confocal microscope. For consistency, confocal stacks were captured under identical settings for each experimental set. Adobe Photoshop and Adobe Illustrator CS3 were utilized solely for image assembly, ensuring that no adjustments were made to the fluorescence intensity.*

*To ensure randomness and objectivity in our image selection, esg-GFP positive cells were selected randomly from the R4 region of the Drosophila midgut. Regions of Interest (ROIs) were also randomly chosen, with an area of 1.5 x 10^4^ mM^2^ from the R4 region. Ten esg-GFP+ cells per ROI were random chosen for quantification. The quantification of midgut cells was conducted using a Leica DM6-B microscope. The esg-GFP positive cell were randomly selected from R4 region of Drosophila midgut.*

*For the analysis of immunofluorescence imaging results from the z stacks acquired with confocal microscopy, we employed ImageJ software, following the methodology previously described by Martin Fitzpatrick, which can be found at the following link:* [*https://theolb.readthedocs.io/en/latest/imaging/measuring-cell-fluorescence-using-imagej.html*](https://theolb.readthedocs.io/en/latest/imaging/measuring-cell-fluorescence-using-imagej.html)*.*

*We hope this detailed explanation addresses your concerns and provides the necessary transparency regarding our image selection and quantification processes. We are committed to maintaining the highest standards of research integrity.*

4. The data in Figures 1K-L suggest an intriguing possibility: that CoA restrains ISC proliferation (and gut aging) via a cell-autonomous mechanism or by preventing intestinal barrier disruption, which has been shown to cause ISC hyperproliferation in previous studies. This distinction needs further investigation.

*Response:*

*Thank you for highlighting the intriguing possibility suggested by the data in Figures 1K-L regarding the role of CoA in restraining ISC proliferation and gut aging. We appreciate your insight and agree that this distinction warrants further investigation.*

*To address your suggestion, we have conducted additional experiments by knockdown dPANK/fbl specifically in enterocytes (ECs) using the mex-GAL4 driver and* *in* *enteroendocrine cells (EEs) using pros-GAL4. Our observations indicate that driven dPANK/fbl RNAi in ECs or EEs did not significantly affect ISC proliferation or barrier function. These findings lead us to conclude that CoA's inhibitory effect on ISC proliferation is mediated through a cell-autonomous mechanism rather than by preventing intestinal barrier disruption.*

*Supplementary Fig. 4 A-G*

*We have incorporated these results into the revised manuscript to provide a clearer understanding of the mechanisms by which CoA exerts its effects. We believe this additional data strengthens our conclusions and addresses the important point you raised.*

*We are grateful for your valuable feedback and for the opportunity to further substantiate our findings.*

5. The authors should examine whether dPANK/fbl overexpression increases CoA levels in the gut (Figure 3C) and whether dPANK/fbl overexpression prevents ISC proliferation in aging guts.

*Response:*

*We are deeply grateful for your thoughtful suggestion regarding the examination of dPANK/fbl overexpression's impact on CoA levels in the gut and its potential preventative effect on ISC proliferation in aging guts.*

*In response to your valuable comment, we have conducted further experiments. We discovered that overexpression of dPANK/fbl (OE) using esg-GAL4 in the midguts of aged flies, increased CoA levels (Supplementary Fig. 1 C).*

*Supplementary Fig. 1 C*

*In alignment with the effects observed upon CoA administration, dPANK/fbl OE with esg-GAL4 showed significantly reduced in ISC proliferation and esg or NRE positive progenitor cells accumulation in midguts from old flies (Supplementary Fig. 2 C-G). These findings support the notion that dPANK/fbl overexpression can prevent age-associated ISC proliferation, which is consistent with the role of CoA administration in this process.*

*Supplementary Fig. 2 C-G*

*We have incorporated these additional results into the revised manuscript, which we trust will address your esteemed concerns and enhance the robustness of our study. We are profoundly thankful for your discerning feedback and for the opportunity to further refine our work.*

6. Fig. 4D, the authors should show the significance between “40d” and “40d+CoA” groups.

*Response:*

*Thank you for your comment. We have added the quantification to* *Figure 4D as requested.*

*Fig. 4D*

7. Fig. 4G-4J, using “eating percentage” to evaluate gut function seems speculative. The authors should provide citations to support this as a reliable marker.

*Response:*

*Thank you for your valuable feedback on the use of "eating percentage" as an indicator of gut function in Figures 4G-4J.*

*We appreciate your concern and have taken the liberty to provide further clarification. The "Eating" status of flies is determined by the ingestion of food containing bromophenol blue, which stains their guts blue. Conversely, "non-eating" flies have not consumed the dyed food and thus show no blue staining. We define "Eating (%)" as the percentage of guts that are stained blue, which serves as our metric for gut function.*

*This method is supported by previous studies, as referenced in [10, 11], where similar methodologies have been employed to assess gut function. In the context of these studies, "Perturbed A" indicates a loss of the acidic region, leading to a basic pH throughout the gut; "Perturbed B" signifies a loss of the strongly acidic region, with the remainder of the gut becoming less acidic (weak blue). In our manuscript, we have defined "Perturbed B" as non-eating, as bromophenol blue is not observed in the midgut of these flies.*

*We trust that this explanation provides the necessary context and validation for using "eating percentage" as a reliable marker for gut function in our study.*

*(source data from Li H et al, Cell Host Microbe, 2016)*

8. There is only one example of lifespan extension (Fig 4K). Since lifespan extension is a major point of the paper, more lifespan data should be included. Examples from both sexes, with different levels of CoA and Pantothenate supplementation could be provided. This lifespan data should be extensive and conclusive. Please also address the relatively modest effects of CoA on lifespan in Figure 4.

*Response:*

*Thank you for your feedback. We have added additional lifespan data for both sexes with different CoA and pantothenate levels in Figures 4B, 6A, and 7C as requested. our observations indicated that lifespan extension of lifelong CoA administration can vary with concentration and was not associated with sex (Figs 4 K and 4L, and S3A, S3B). And we found 0.5mM CoA was effective on extend lifespan compared with old WT flies. However, a higher CoA (2.5mM) concentration could not further extend lifespan. Moreover, we also investigated whether Pan administration could extend lifespan. We found 1mM Pan was effective to prolong lifespan (S3A, S3B Fig).*

*In response to your concerns about the modest effects of CoA on lifespan as shown in Figure 4, we acknowledge that lifespan is influenced by a multitude of factors, and CoA is just one of them. Therefore, the increase in lifespan observed after CoA supplementation is not as pronounced as it might be if CoA were the sole determinant of longevity.*

*Fig. 4K-L*

*Supplementary Fig. 3 A-B*

9. The figure legends need to be revised to provide clear, concise, and complete descriptions of the data. Crucially, any experimental details missing in the Results section should be included in the figure legends to ensure that the figures are independently interpretable. For example, details about specific controls, experimental conditions, or quantification methods should be described in the legends if omitted elsewhere in the manuscript.

*Response:*

*Thank you for your feedback. We have revised the figure legends to be more clear and complete, ensuring all necessary experimental details are included.*

10. Please elaborate on the mechanistic link between CoA’s antioxidative properties and its ability to modulate ISC proliferation and differentiation.

*Response:*

*We appreciate this suggestion. We have expanded on our hypothesis in the revised manuscript. We propose that the reduction of CoA in ISCs from aged flies leads to iron accumulation. This iron overload subsequently results in mitochondrial damage and the generation of reactive oxygen species (ROS)[12].* *It is well-established that ROS can activate the c-Jun N-terminal kinase (JNK) pathway. The sustained activation of JNK in stem cells is known to cause overproliferation of ISCs and differentiation defects in their progeny**[5], which ultimately contributes to the age-related deterioration of the intestinal epithelium.*

*We believe this clarification strengthens the mechanistic understanding of how CoA modulates ISC behavior and addresses your query.*

Minor points:

1. In the introduction, it would be helpful to review previous studies examining whether defects in the CoA biosynthesis pathway correlate with colorectal cancer progression.

*Response:*

*We appreciate your valuable suggestions. In response, we have updated the introduction in the revised manuscript to include a discussion on the association between CoA biosynthesis and colorectal cancer. Specifically, we have added the following points:*

*"Primary colon adenocarcinoma contains significantly (P < 0.001) more pantothenate than normal colon tissue**[13]. It is also observed disruptions in the pantothenate and CoA biosynthesis pathways in the Colorectal Cancer (CRC) group**[14]. Furthermore, it has been demonstrated that TMEM120A's nuclear localization is crucial for its role in regulating CRC chemosensitivity. Mechanistically, the nuclear subpopulation of TMEM120A plays a key role in sustaining nuclear CoA levels, which in turn influences the levels of nuclear acetyl-CoA and histone acetylation in CRC cells* *[15]."*

2. Also in the introduction, the authors mentioned that “using a screening strategy, we found that the pantothenate/coenzyme A (CoA) pathway is abundant in young ISCs and identified CoA as an inhibitor of age-related ISC over-proliferation.” Please provide a little bit more details about this screen in the Method section.

*Response:*

*Thank you for your suggestion to provide more details about the screening strategy mentioned in the introduction.*

*In the revised manuscript, we have expanded the Methods section to include a detailed description of our screening method. Here is the additional information we have provided:*

*"To elucidate the regulation of age-associated functional decline of ISCs by endogenous small molecules, we screened for small natural compounds that could repress age-related accumulation of esg+ cells in Drosophila midguts. We utilized an 'esg-luciferase' reporter system, which is based on luciferase reporter expression driven by esg-GAL4 and UAS-luciferase in Esg+ cells. This system allows for the tracing and quantification of real-time changes in ISCs and their differentiating progenies within aging Drosophila midguts.*

*Luciferase activity was measured using the Firefly Luciferase Reporter Gene Assay Kit (Beyotime Biotechnology, Jiangsu, China, RG051S). For each sample, fifteen female adult midguts were dissected in cold PBS and immediately frozen in liquid nitrogen. Samples were then homogenized in 50μl of Luciferase Reporter Gene Assay Lysis Buffer provided by the manufacturer. Extracts were centrifuged at 13,000 g for 10 minutes at 4℃ to obtain samples for testing. Samples were collected over a series of days and stored at -80℃ until six independent samples were collected for each genotype. Luciferase activity was assessed following the manufacturer's protocols."*

*We hope that this detailed description sufficiently addresses your request and provides the necessary transparency into our screening methodology.*

3. Please define ROIs and the number of regions analyzed for quantification, particularly in figures that rely on spatial specificity (e.g., Figures 1J, 3E, 5D, 6D).

*Response:*

*We appreciate your valuable suggestions. We have defined ROIs and specified the number of regions analyzed in the figure legends for the relevant figures.*

4. Please reduce nonspecific background staining in image-based quantifications and explain the steps to ensure reliable measurements.

*Response:*

*Thank you for your suggestions. We apologize for not providing a clear description earlier regarding the fluorescence intensity measurements. We would like to clarify that background subtraction was performed during the analysis. The specific method for quantifying fluorescence intensity is as follows:*

*Immunofluorescence imaging results were analyzed based on z stacks acquired with confocal microscopy. The fluorescence intensity of the region of interest (ROI) or cell was calculated using ImageJ software, following the method described by Martin Fitzpatrick at the website: https://theolb.readthedocs.io/en/latest/imaging/measuring-cell-fluorescence-using-imagej.html. The detailed steps are as follows:*

*1. Open image: File -> Open.*

*2. Split Channels: Image -> Color -> Split Channels. Keep the channel to be calculated and delete other channels.*

*3. Set scale to ensure the unit of length is in Pixels: Analyze -> Set Scale -> click to remove scale.*

*4. Set measurements: Analyze -> Set Measurements. Tick the boxes marked “Area”, “Integrated Density”, and “Limit to Threshold”.*

*5. Click on the “OK” button.*

*6. Select the ROI or cell using any of the drawing/selection tools and select a smaller region around the ROI or cell as background.*

*7. Select different ROI or cell and background regions by using the ROI Manager.*

*8. Tick the boxes marked “Measure” to calculate the integrated density.*

*9. The final calculation is performed as follows:* *Integrated Density = (Integrated Density of ROI or cell - Integrated Density of background region) / (Area of background region × Area of ROI or cell).*

*We hope this detailed explanation clarifies our approach to fluorescence intensity analysis.*

5. Please provide detailed information on the ages of flies used in all experiments, especially in Figures 3C and 3D.

*Response:*

*Thank you for your valuable suggestions. We have included detailed information regarding the ages of the flies used in all experiments, with specific emphasis on Figures 3C and 3D, in the revised manuscript.*

*Supplementary Fig. 1C, 1D*

6. The abbreviation “FAC” should be defined where it is first introduced (page 9).

*Response:*

*Thank you for your suggestion. We have defined the abbreviation "FAC" (iron salt ferric ammonium citrate, an iron supplement) where it is first introduced on page 9 in the revised manuscript.*

**Reply to Reviewer #3:**

Reviewers' Comments:

Reviewer #3: Drosophila intestinal stem cells (ISCs) represent a powerful model to elucidate the mechanisms controlling and executing regeneration associated with aging fueled by somatic stem cells. In their manuscript ‘Age-associated Decline of Coenzyme A leads to Intestinal stem cells dysfunction via disturbing iron homeostasis’, The work described by Liu and colleagues identified and report Coenzyme A(CoA) as a key driver in regenerative and aging associated dysfunction of the adult Drosophila midgut with a screening approach. Furthermore, they show that the CoA regulated aged ISC dysfunction through regulating ISC iron availability. It represents a significant gain in the understanding of somatic stem cell-driven tissue repair and regeneration in the fly gut. The described metabolic mechanism may operate similarly in other examples of stem cell function and, more generally, in diverse examples of aging associated tissue dysfunction. The manuscript is well written and data is comprehensively presented. The data is based on state-of-the-art methods and provides new important insights in the mechanisms of stem cell differentiation and tissue regeneration associated with aging. General interest is high. Publication as a *PLOS Genetics* paper is appropriate.

Although the authors present data on their findings, I have a few suggestions and concerns that I think should be approached experimentally and discussed in more detail in a revised version of this manuscript.

*Response:*

*We sincerely appreciate your favorable evaluation and the positive remarks of our manuscript. The comments are exceptionally helpful in enhancing the overall quality of our article. We are committed to addressing each of the concerns you highlighted and have provided a comprehensive response and solution. We hope that the revisions we made as detailed below would address all concerns.*

Minor comment:

1、Could the authors provide speculation on how the CoA regulated iron homeostasis during the fbl is depleted, allowing the ROS to be accumulated?

*Response:*

*Thank you for your insightful question regarding the regulation of iron homeostasis by CoA during dPANK/fbl depletion and its association with ROS accumulation.*

*We acknowledge that the exact mechanisms of iron accumulation in ISCs following dPANK/fbl loss are yet to be determined. However, we can infer from studies on neurodegeneration associated with PANK2 loss that the absence of active enzyme may lead to a buildup of substrates in the CoA biosynthetic pathway, particularly N-pantothenyl cysteine and free cysteine[2, 3]., which can chelate iron and lead to its accumulation.*

*Furthermore, the localization of PANK2 at the mitochondria prompted several groups to investigate mitochondrial dysfunction in PKAN pathogenesis. Notably, the significant findings by Paolo Santambrogio et al., [4] identified defects in tubulin acetylation/phosphorylation in PKAN astrocytes. Thus, we speculate that this could lead to unregulated vesicular dynamics and incorrect iron delivery to mitochondria, resulting in iron mislocalization. This scenario could cause cytosolic iron overload and mitochondrial iron deficiency, potentially triggering mitochondrial dysfunction and ROS accumulation.*

*We hypothesize that a similar mechanism might be at play in the context of dPANK/fbl and iron accumulation in ISCs, and we intend to explore this possibility in future studies.*

2、The sequence of the figures is not intuitive and could be confusing for readers. Please consider rearranging the order of the figures for better clarity. For instance, figures 2F-2H should be moved to the left. Additionally, it is recommended to place statistical graphs on the right side.

*Response:*

*We apologize to the reviewer for any confusion caused by the data presentation. We have reorganized the figures in the revised manuscript for better clarity.*

3、Reference to previous work on has emerged as a potential strategy for enhancing stem cell functionality (4). The reference cited is not of an earlier publication; please search for and cite more earlier literature account of metabolism in stem cells.

*Response:*

*Thank you for your suggestion. We have located and included additional earlier literature on metabolism in stem cells in the revised manuscript.*

4、In Figure 3, to test whether fbl RNAi knockdown in ISCs of young fruit flies would result in a stem cell aging phenotype, the authors employed only one fbl RNAi line to examine the phenotype following knockdown in stem cells. Is there any possibility that off-target effects from the RNAi could be responsible for this? Some of the central conclusions made in the study do require additional data to be substantiated.

*Response:*

*Thank you for your suggestion. We have added data from another fbl RNAi line to ensure the robustness of our findings, and the results are consistent with our previous conclusions, mitigating concerns about off-target effects.*

*Supplementary Fig. 2 A-B*

5、This figure has hardly any esg-GFP+ cells signals in the 14 day condition. While the number esg-GFP+ cells of flies with fbl knockdown or 40 day is robust, the absence of esg-GFP+ looks weird. Please comment, and add a description in the text.

*Response:*

*We agree the GFP signal is weak in the ad 14- day flies (although its present). Admittedly, some of the GFP signal is ‘drowned out’ by DAPI. However, in homeostatic conditions, it is normal to see very few esg-GFP+ cells (ISCs and EBs) in a healthy midgut. This is further confirmed by our quantification of Delta positive cells in 14-day flies, which are very low.*

6、Figure 5A: why does the ferric iron signal have a dotted pattern of young flies with fbl knockdown in Figure 5, and its signal is not observed in young flies? This is also partially observed in the old flies (Fig 5E).

*Response:*

*Thank you for your observation regarding the ferric iron signal in Figure 5A and its pattern in young flies with fbl knockdown, as well as its partial observation in old flies (Fig 5E). We have investigated this matter and found that the dotted pattern is due to the cells limiting the size of the intracellular labile iron pool (LIP)[16, 17], which constitutes a small percentage of the total cellular iron and helps curb Fenton-reaction-mediated ROS generation[18, 19]. The mechanisms that define LIP size are not fully understood but may stem from a balance of iron demand, storage capacity, and local availability. Any disruption in LIP size quickly activates cellular iron homeostasis mechanisms, such as the iron response that restores LIP when iron is insufficient[19, 20].*

*We were not previously aware of changes in LIP size or iron homeostasis pathway activation during cell division, particularly in the context of hematopoiesis. This observation in our study has prompted further investigation into these dynamics, which we believe is a valuable contribution to the understanding of iron homeostasis in cellular contexts.*

**Reference**

1.	Du G, Qiao Y, Zhuo Z, Zhou J, Li X, Liu Z, et al. Lipoic acid rejuvenates aged intestinal stem cells by preventing age-associated endosome reduction. EMBO Rep. 2020;21(8):e49583.

2.	Zhou B, Westaway SK, Levinson B, Johnson MA, Gitschier J, Hayflick SJ. A novel pantothenate kinase gene (PANK2) is defective in Hallervorden-Spatz syndrome. Nat Genet. 2001;28(4):345-9.

3.	Perry TL, Norman MG, Yong VW, Whiting S, Crichton JU, Hansen S, et al. Hallervorden-Spatz disease: cysteine accumulation and cysteine dioxygenase deficiency in the globus pallidus. Ann Neurol. 1985;18(4):482-9.

4.	Santambrogio P, Cozzi A, Balestrucci C, Ripamonti M, Berno V, Cammarota E, et al. Mitochondrial iron deficiency triggers cytosolic iron overload in PKAN hiPS-derived astrocytes. Cell Death Dis. 2024;15(5):361.

5.	Biteau B, Hochmuth CE, Jasper H. JNK activity in somatic stem cells causes loss of tissue homeostasis in the aging Drosophila gut. Cell Stem Cell. 2008;3(4):442-55.

6.	Tauc HM, Rodriguez-Fernandez IA, Hackney JA, Pawlak M, Ronnen Oron T, Korzelius J, et al. Age-related changes in polycomb gene regulation disrupt lineage fidelity in intestinal stem cells. Elife. 2021;10.

7.	Salle J, Gervais L, Boumard B, Stefanutti M, Siudeja K, Bardin AJ. Intrinsic regulation of enteroendocrine fate by Numb. EMBO J. 2017;36(13):1928-45.

8.	Patel PH, Penalva C, Kardorff M, Roca M, Pavlovic B, Thiel A, et al. Damage sensing by a Nox-Ask1-MKK3-p38 signaling pathway mediates regeneration in the adult Drosophila midgut. Nat Commun. 2019;10(1):4365.

9.	Liu F, Zhao H, Kong R, Shi L, Li Z, Ma R, et al. Derlin-1 and TER94/VCP/p97 are required for intestinal homeostasis. J Genet Genomics. 2022;49(3):195-207.

10.	Li H, Qi Y, Jasper H. Preventing Age-Related Decline of Gut Compartmentalization Limits Microbiota Dysbiosis and Extends Lifespan. Cell Host Microbe. 2016;19(2):240-53.

11.	Abu F, Ohlstein B. Monitoring Gut Acidification in the Adult Drosophila Intestine. J Vis Exp. 2021(176).

12.	Huang Y, Wan Z, Tang Y, Xu J, Laboret B, Nallamothu S, et al. Pantothenate kinase 2 interacts with PINK1 to regulate mitochondrial quality control via acetyl-CoA metabolism. Nat Commun. 2022;13(1):2412.

13.	Baker H, Frank O, Chen T, Feingold S, DeAngelis B, Baker ER. Elevated vitamin levels in colon adenocarcinoma as compared with metastatic liver adenocarcinoma from colon primary and normal adjacent tissue. Cancer. 1981;47(12):2883-6.

14.	Yi Y, Wang J, Liang C, Ren C, Lian X, Han C, et al. LC-MS-based serum metabolomics analysis for the screening and monitoring of colorectal cancer. Front Oncol. 2023;13:1173424.

15.	Wang L, Liu X. TMEM120A-mediated regulation of chemotherapy sensitivity in colorectal cancer cells. Cancer Chemother Pharmacol. 2024;93(1):11-22.

16.	Kao YR, Chen J, Kumari R, Ng A, Zintiridou A, Tatiparthy M, et al. An iron rheostat controls hematopoietic stem cell fate. Cell Stem Cell. 2024;31(3):378-97 e12.

17.	Jacobs A. An intracellular transit iron pool. Ciba Found Symp. 1976(51):91-106.

18.	Rothman RJ, Serroni A, Farber JL. Cellular pool of transient ferric iron, chelatable by deferoxamine and distinct from ferritin, that is involved in oxidative cell injury. Mol Pharmacol. 1992;42(4):703-10.

19.	Kruszewski M. Labile iron pool: the main determinant of cellular response to oxidative stress. Mutat Res. 2003;531(1-2):81-92.

20.	Kakhlon O, Cabantchik ZI. The labile iron pool: characterization, measurement, and participation in cellular processes(1). Free Radic Biol Med. 2002;33(8):1037-46.
